# Growth of wood-inhabiting yeasts of the Faroe Islands in the presence of spent sulphite liquor

**DOI:** 10.1007/s10482-021-01543-5

**Published:** 2021-04-13

**Authors:** Jonas Rönnander, Sandra Ann Ingela Wright

**Affiliations:** Faculty of Engineering and Sustainable Development, University of Gävle, 80176 Gävle, Sweden

**Keywords:** Inhibitor, Lignin, Lignocellulosic hydrolysate, Lignosulfonate, Psychrotolerant, SSL

## Abstract

In the microbial community of decaying wood, yeasts are important for the recycling of nutrients. Nevertheless, information on their biodiversity in this niche in the Northern hemisphere is limited. Wood-colonising yeasts encounter identical and similar growth-inhibitory compounds as those in spent sulphite liquor (SSL), an energy-rich, acid hydrolysate and waste product from the paper industry, which may render them well-suited for cultivation in SSL. In the present study, yeasts were isolated from decaying wood on the Faroe Islands and identified based on sequence homology of the ITS and D1/D2 regions. Among the yeasts isolated, *Candida argentea*, *Cystofilobasidium infirmominiatum*, *Naganishia albidosimilis*, *Naganishia onofrii*, *Holtermanniella takashimae* and *Goffeauzyma gastrica* were new to decaying wood in cold and temperate climates. *C. argentea* and *Rhodotorula* are rarely-isolated species, with no previous documentation from cold and maritime climates. The isolates were further tested for growth in a medium with increasing concentrations of softwood SSL. Most grew in the presence of 10% SSL. Isolates of *Debaryomyces* sp*.*, *C. argentea* and *Rhodotorula* sp. were the most tolerant. Representatives of *Debaryomyces* and *Rhodotorula* have previously been found in decaying wood. In contrast, the least tolerant isolates belonged to species that are rarely reported from decaying wood. The relative importance of individual inhibitors to yeast growth is discussed. To our knowledge, none of the present yeast species have previously been cultivated in SSL medium. Decaying wood can be a useful future source of yeasts for valorisation of various hydrolysates to industrial chemicals and biofuels.

**Supplementary Information:**

The online version of this article (10.1007/s10482-021-01543-5) contains supplementary material, which is available to authorized users.

## Introduction

Wood-decaying microorganisms play a key role in the recycling of nutrients in forest ecosystems. Fungi are considered the principal decomposers in terrestrial systems and are responsible for the three major visual categories of wood rot: white-rot, brown-rot and soft-rot. Natural wood decay involves a consortium of different organisms (Stokland 2012). Through the action of white-rot fungi, many components of wood become accessible to yeasts, bacteria and filamentous fungi for further mineralization (Paliwal et al. 2012). During different stages of wood decay, the composition of the yeast microflora changes (González et al. 1989). A review of the literature on yeasts in decaying wood from cold and temperate regions reveals a wealth of ascomycetous and basidiomycetous yeasts (Supplementary Tables S1a−b). The relative amounts of the three major constituents of wood: cellulose, hemicellulose and lignin vary immensely among different plant species and types of lignocellulosic materials (Henriksson et al. 2009; Stokland 2012). Yeasts that colonise decaying wood and leaf debris assimilate the monosaccharides resulting from the depolymerisation of polysaccharides; initially, those from hemicellulose and pectin, and subsequently, those from cellulose, as reviewed by Cadete et al. (2017). The lignin component of wood is highly recalcitrant to biodegradation and consists of small monoaromatic units, which are relatively inaccessible and also growth-inhibitory to microorganisms. However, there are yeasts that can metabolise this class of compounds (Middelhoven 1993; Sampaio 1999). By detoxifying these phenolic compounds, yeasts may create an environment that is conducive also to other wood-degrading microorganisms.

In contrast to natural decay, chemical and mechanical treatment is used to artificially prepare wood for paper production or biotechnological processes. Wooden hydrolysates are obtained through a range of different hydrolytic treatment methods (Parajó et al. 1998). Spent sulphite liquor (SSL) is one type of acid hydrolysate, which is produced as a waste product in the sulphite paper pulping process. This process involves the heating of wooden chips in the presence of sulphur dioxide (SO_2_) and water. Bisulphite ions are generated, which react with the lignin polymer, and through this process, the wood is delignified and lignosulphonates form (Evtuguin 2016). Three categories of substrates can be used for the production of SSL: hardwood (e.g. willow, poplar, birch and red oak), softwood (e.g. spruce and pine) and herbaceous material (e.g. sugarcane bagasse, corn stover and wheat straw). The type of substrate has a bearing on the relative amounts of lignin, cellulose and hemicellulose, and their respective degradation products present in SSL (Klinke et al. 2004; Richardsson et al. 2011).

Yeasts that are cultivated in hydrolysates encounter three classes of growth-inhibitory compounds. These are furan aldehydes (furfural and 5-(hydroxymethyl)furfural (5-HMF)), aliphatic acids (e.g. formic acid and acetic acid) and monoaromatic phenolic compounds (Adeboye et al. 2014). Monosaccharides, i.e. hexose and pentose sugars, are generated through acid hydrolysis of cellulose and hemicellulose, respectively (Evtuguin 2016; Pereira et al. 2013). The monosaccharides are successively converted to aldehydes and aliphatic acids. The furan aldehydes, furfural and 5-HMF, are formed from pentose and hexose sugars, respectively (Jönsson et al. 2013). The furan aldehydes act as inhibitors by interfering with several glycolytic enzymes, such as hexokinase, glyceraldehyde-3-phophate dehydrogenase and alcohol dehydrogenase (Richardsson et al. 2011). Acetic acid (p*K*a 4.76 at 20 °C) and formic acid (p*K*a 3.75 at 20 °C) inhibit cells in a similar manner. Their undissociated forms accumulate inside yeast cells, where they dissociate and release protons due to the higher intracellular pH (7.0–7.2). In order to maintain homeostasis, excess intracellular protons are subsequently exported through the cellular membrane, resulting in the depletion of ATP and ensuing growth inhibition (Almeida et al. 2007; Richardsson et al. 2011). Examples of monoaromatic phenolic compounds that originate from acid hydrolysis of lignin in softwood are: vanillin, vanillic acid, ferulic acid, benzoic acid, 3,4-dihydroxybenzoic acid, 4-hydroxybenzaldehyde, dihydroxyconiferyl alcohol, coniferyl aldehyde, syringaldehyde and syringic acid (Du et al. 2010; Larsson et al. 1999). The inhibitory activity of these compounds depends on several variables, such as concentration, the nature of the functional groups and substituent positions (Almeida et al. 2007).

SSL is a cheap resource, attractive for biotechnological applications. The monosaccharides and the energy-rich lignin in SSL and other hydrolysates can be used as substrates for the microbial production of xylitol, arabinitol, bioethanol, lipids for biodiesel, single cell proteins, vanillin, vanillic acid, ferulic acid and plastic monomers (Brethauer and Studer 2015; Breuer and Harms 2006; Parajó et al. 1998). Liquid medium supplemented with softwood SSL has been used for the production of ethanol and xylitol through fermentation by various yeast species of the Saccharomycetes and Schizosaccharomycetes, as reviewed by Weissgram et al. (2015). A strain of *Scheffersomyces stipitis* (at the time known as *Candida stipitis*) was cultivated in 60% undetoxified hardwood SSL, whose glucose and xylose was fermented to ethanol and xylitol, respectively (Pereira et al. 2015). Yeasts that naturally reside on decaying wood encounter many of the inhibitors and monosaccharides present in hydrolysates. In fact, decaying rainforest wood has been used for isolation of yeasts that subsequently were successfully cultivated in sugarcane bagasse hydrolysate (Guamán-Burneo et al. 2015; Morais et al. 2020). With a few exceptions (Middelhoven 2006; Péter et al. 2003; Sorenson et al. 1991), yeast isolations from decaying wood have for the most part taken place in the Southern Hemisphere (Cadete et al. 2017), and none have been conducted on decaying wood in Nordic countries, for example on the Faroe Islands, where the climate is classified as subarctic oceanic (Cfc) (Kottek et al. 2006). In addition, very little is known about the yeast communities on these islands. In contrast, the yeast flora on islands, such as the Galapagos Islands, the King George Islands, the South Shetlands and the Arctic Svalbard Islands has recently been investigated (Guamán-Burneo et al. 2015; Perini et al. 2019; Rovati et al. 2013; Troncoso et al. 2017). Faroese decaying wood could harbour novel yeast candidates with promise for cultivation in softwood SSL, which is based on spruce and pine trees, species that prevail in Nordic countries.

The aim of the present study was to isolate and characterise yeasts associated with decaying wood on the Faroe Islands. The study also addresses the tolerance of these yeasts to SSL. Like other wooden hydrolysates, SSL contains many of the components present in decaying wood and has only rarely been utilised for yeast cultivation studies.

## Materials and methods

### Sampling and isolation of yeasts

**Fig. 1:**
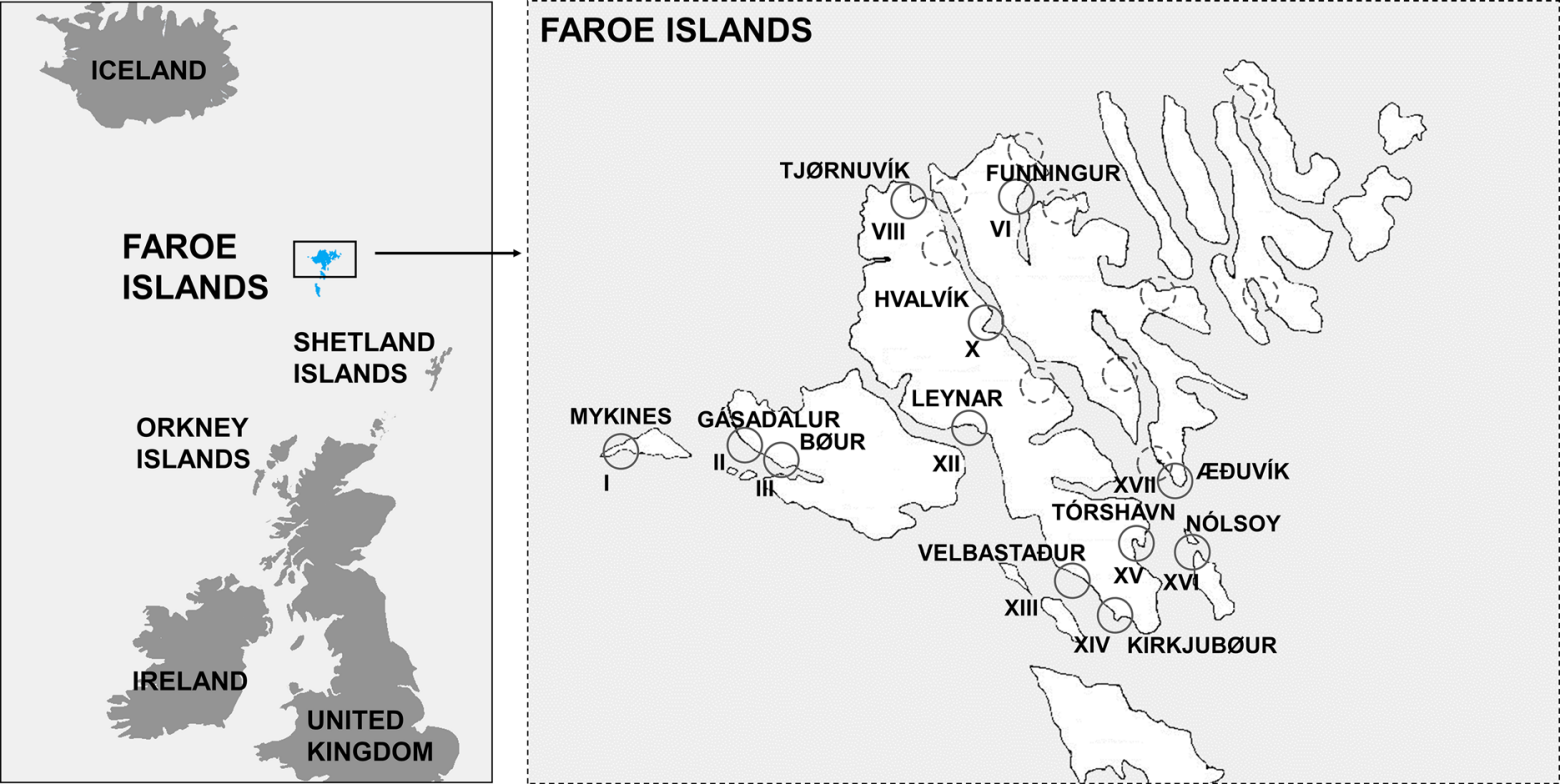
Locations on the Faroe Islands from where yeasts were isolated from sampled wooden chips, indicated by circles and Roman numerals. Dashed circles indicate locations where no yeasts were isolated. A complete list of locations sampled is presented in Supplementary Table S2; GPS coordinates are indicated in Table 1. The map to the left is based on an excerpt from Nordic Countries including Estonia.jpg by Jaan-Matti Saul, alias Blomsterhagens CC BY-SA 4.0

Sixty-eight wooden chips of approximately 10 mm length were excised from walls and foundations of weathered buildings, fences and planks on seven of the 18 islands, which constitute the Faroe Islands. The decaying wood sampled sometimes had soil adhering to it. The chips were placed in individual Eppendorf tubes, which contained 0.5 ml of sterile liquid lignin modifying enzyme basal medium (LBM). LBM is often used as a basal medium for dissolving lignin (Pointing 1999). Sampling took place from the 9th to the 12th of July, 2014, when the mean temperature in Tórshavn was 10.8 °C and the mean relative humidity was high (91%) (Supplementary Fig. S1). From five of the islands, isolates were recovered. Each geographical location was designated a Roman numeral, and GPS data were recorded (Table 1, Supplementary Table S2; Fig. 1). After sampling, the wooden chips were maintained at 8 °C in LBM for a period of 7–11 days. The chips were subsequently transferred with sterile tweezers to individual tubes, each containing 1 ml of sterile tap water. The wood was ground with a glass rod for one minute and the resulting suspension was diluted 1:100 in sterile tap water. After vortexing, aliquots of 100 µl of diluted suspensions were plated on yeast extract peptone dextrose (YPD) medium (Zimbro et al. 2003), supplemented with 20.0 g bacteriological agar l^–1^ and antibiotics, as previously described (Rönnander et al. 2018). The yeast isolates were deposited in the culture collection at the University of Gävle, Sweden (UGCC) and maintained at –80 °C in a medium consisting of 15% glycerol in liquid YPD medium (Supplementary Table S3). For all experiments, yeasts were routinely cultured on YPD medium at 20 °C.

**Table 1:** Yeasts isolated from wooden samples from several locations on the Faroe Islands, and reference strains

Phylum	Isolate	Species^a^	Origin				GenBank accession no		References^d^
			Local community	GPS coordinates		Location	D1/D2	ITS	
			Country^b^	Latitude	Longitude	No.^c^			
Ascomycota	FTJA004	*Debaryomyces* sp.	Tjørnuvík, FO	62° 17′ 17″ N	7° 8′ 27″ W	VIII	–	MK749417.1	This work
	FLYA002	*Debaryomyces* sp.	Leynar, FO	62° 6′ 58″ N	7° 2′ 21″ W	XII	MK749768.1	MK737678.1	This work
	FAEA002	*Nadsonia. starkeyi-henricii*	Æðuvík, FO	62° 4′ 11″ N	6° 41′ 24″ W	XVII	MK749675.1	MK737060.1	This work
	FAEA004	*Nadsonia starkeyi-henricii*	Æðuvík, FO	62° 4′ 11″ N	6° 41′ 24″ W	XVII	MK749674.1	MK737084.1	This work
	FVE002	*Candida sake*	Velbastaður, FO	61° 59′ 5″ N	6° 51′ 2″ W	XIII	MK749934.1	MK749435.1	This work
	FNOA002	*Candida sake*	Nólsoy, FO	62° 0′ 33″ N	6° 40′ 7″ W	XVI	MK749932.1	–	This work
	FGAA004	*Candida argentea*	Gásadalur, FO	62° 6′ 44″ N	7° 26′ 5″ W	II	MK749751.1	MN010514.1	This work
Basidiomycota	FMYE002	*Cystobasidium laryngis*	Mykines, FO	62° 6′ 0″ N	7° 36′ 0″ W	I	MK749841.1	MK737743.1	This work
	FMYD002	*Cystobasidium laryngis*	Mykines, FO	62° 6′ 0″ N	7° 36′ 0″ W	I	MK749842.1	MG674823.1	(Rönnander et al. 2018)
	FTOF002	*Cystobasidium laryngis*	Tórshavn, FO	62° 0′ 42″ N	6° 46′ 3″ W	XV	MK749935.1	MK749434.1	This work
	FHVB002	*Cystobasidium laryngis*	Hvalvík, FO	62° 11′ 23″ N	7° 1′ 51″ W	X	MK749766.1	MK737645.1	This work
	FKIA004	*Cystofilobasidium infirmominiatum*	Kirkjubøur, FO	61° 57′ 22″ N	6° 47′ 37″ W	XIV	MK749769.1	MK737652.1	This work
	FTJA008	*Cystofilobasidium infirmominiatum*	Tjørnuvík, FO	62° 17′ 17″ N	7° 8′ 27″ W	VIII	MK749931.1	MK749425.1	This work
	FMYH004	*Goffeauzyma gastrica*	Mykines, FO	62° 6′ 0″ N	7° 36′ 0″ W	I	MK749864.1	MK737745.1	This work
	FBOB002	*Goffeauzyma gastrica*	Bøur, FO	62° 5′ 27″ N	7° 22′ 3″ W	III	MK749731.1	MK737521.1	This work
	FXXA004	*Naganishia albidosimilis*	FO	–	–	VI	MK749999.1	MK749445.1	This work
	FMYH004b	*Naganishia onofrii*	Mykines, FO	62° 6′ 0″ N	7° 36′ 0″ W	I	MK749865.1	MK737744.1	This work
	FBOC004	*Holtermanniella takashimae*	Bøur, FO	62° 5′ 27″ N	7° 22′ 3″ W	III	MK749752.1	MK737520.1	This work
	FMYH002b	*Rhodotorula* sp.	Mykines, FO	62° 6′ 0″ N	7° 36′ 0″ W	I	MK749855.1	MK737742.1	This work
Reference strains^e^									
Ascomycota	CBS11284^T^	*Ogataea thermophila*	Goryung, KR	–	–	–	KY102443.1	KY102443.1	(Shin et al. 2001)
Basidiomycota	CBS2221^T^	*Cystobasidium laryngis*	NO	–	–	–	KY107432.1	KY103134.1	(Reiersøl 1955)
	CBS10436^T^	*Phenoliferia glacialis*	Innsbruck, AU	46° 59′ 12″ N	11° 69′ 53″ E	–	KY108773.1	KY104503.1	(Margesin et al. 2007)

^a^Identification was based on closest BLAST match from BLASTN searches of the ITS and D1/D2 regions; Sequence gaps and percentage identity are compiled in Supplementary Table S4; Phylogenetic trees of *G. gastrica, H. takashimae*, *Nag. albidosimilis*, *Nag. onofrii, Debaryomyces* sp. (closest to *D. hansenii*) and *Rhodotorula* sp. (closest to *R. araucariae*) are presented in Supplementary Figs. S2a–d and S4a–b

^b^FO Faroe Islands, KR Korea, NO Norway, AU Austria

^c^The Roman numerals correspond to specific geographical locations, as indicated in Fig. 1. Roman numerals that are missing from the present table represent locations from which no isolates were retrieved. The complete list of sampled locations is presented in Supplementary Table S2; Accession numbers of D1/D2 and ITS sequences for reference strains were retrieved from Genbank

^d^The ITS sequence and its homology was determined for *Cys. laryngis* isolate FMYD002 in Rönnander et al. 2018. The D1/D2 sequence was determined in the present study

^e^The reference strains: *O. thermophila* CBS11284^T^, a thermophilic strain, *Cys. laryngis* CBS2221^T^, a psychrotolerant strain and *Phe. glacialis* CBS10436^T^, a psychrophilic strain were utilised for the growth temperature preference experiment. *Cys. laryngis* CBS2221^T^ was also used in the biotolerance experiments

– = No information available/Not Determined

### Identification of yeasts

Genomic DNA extraction and PCR amplification were carried out for all isolates, according to the protocol used for *Cystobasidium laryngis* FMYD002 (Rönnander et al. 2018). The LSU rDNA D1/D2 region was amplified by using the primers NL1 (5′GCA TAT CAA TAA GCG GAG GAA AAG) and NL4 (5′GGT CCG TGT TTC AAG ACG G), and the ITS1–5.8S–ITS2 region was amplified by using the primers ITS4 (5′TCC TCC GCT TAT TGA TAT GC) and ITS5 (5′GGA AGT AAA AGT CGT AAC AAG G) (White et al. 1990). The same primers were used for sequencing. Consensus sequences of ITS and D1/D2 were constructed by using the Lasergene software package DNAStar® (SeqMan Pro NGen®. Version 14.0. DNASTAR. Madison, WI, USA), and used for searching for sequence homology by using the Basic Local Alignment Search Tool (BLASTN) (Altschul et al. 1990). Results of ITS and D1/D2 sequence comparison that had 1% discrepancy or less to those of type strains were considered the same species (Supplementary Table S4). In cases of ambiguity, phylogenetic trees were generated of separate or concatenated LSU D1/D2 and ITS sequences of the Faroese isolates and close representative members of the genera. Sequences were aligned in MEGA7: Molecular Evolutionary Genetics Analysis version 7.0 for bigger datasets (Kumar et al. 2016), by using MUSCLE (Multiple Sequence Comparison by Log-Expectation) and subjected to Maximum Likelihood molecular phylogenetic analysis, based on the Kimura 2-parameter model. Bootstrap analysis was carried out with 1000 replications. The ITS and D1/D2 sequences were deposited in GenBank (www.ncbi.nlm.nih.gov).

### Growth temperature preferences

Temperature preferences were investigated by monitoring growth on solid culture medium, as described by Kurtzman et al. (2011b), with the modification of using droplets rather than streaking of overnight growth and by using YPD as culture medium. A 3–4-day-old colony of yeast was transferred from YPD solid medium to a 15 ml tube containing 2 ml of YPD broth. After cultivation overnight at 20 °C on a reciprocal shaker at 225 rpm, the cell culture was centrifuged at 3000 × g for 5 min and cells were resuspended in 1.0 ml of sterile tap water. The suspension was diluted 1:50 times in sterile tap water and 5 µl drops were placed on YPD agar. The following strains were used as references and added to each plate: *Ogataea polymorpha* (formerly *Candida thermophila*) CBS11284^T^ (a thermophilic strain), *Cys. laryngis* CBS2221^T^ (a psychrotolerant strain) and *Phenoliferia glacialis* CBS10436^T^ (a psychrophilic strain). The initial culture of the latter strain was cultivated at 13 °C instead of 20 °C, which was the temperature used for the other isolates in this study. Replicate plates were incubated at –3 °C, 4 °C, 15 °C, 20 °C, 25 °C, 30 °C and 37 °C. Growth was assessed visually after 3, 6 and 10 days. Optimal growth temperature range (OTR) was defined as the temperature range between the lowest and the highest temperature that allowed for distinct microbial growth. Based on their temperature profiles, the isolates were classified either as psychrophilic (growth in the range of 0–20 °C; with an optimum at 15 °C) or psychrotolerant (growth at 0 °C; with an optimum in the range of 20–30 °C) (Buzzini and Margesin 2014). The experiment was carried out four times and used two replicates per experiment.

### Preparation of spent sulphite liquor (SSL) and lignosulphonates (LS)

SSL used in the experiments was prepared according to the description below: The 1st batch of SSL consisted of an acid hydrolysate of a softwood chip mixture of approximately 65% spruce and 35% pine (annual average in 2015 for Domsjö fabriker), a liquid which had been prepared by a two-step acid bisulfide batch process that utilised a sodium base. The wood chip mixture was initially adjusted to pH 4.5 and heated to 150 °C in a pressure boiler (400 m^3^). Subsequently, SO_2_ was added. The pH was lowered to 1.5 and the mixture was processed further at 140 °C. The lignosulphonates (LS) used in the experiments were produced from a 2nd batch of SSL, which had been produced in a similar way as described above for the 1st batch. The wooden chips used for the production of the 2nd batch had a wood composition ratio of 70% spruce and 30% pine (October average in 2016 for Domsjö fabriker). LS was prepared by fermenting the accessible monosaccharides dissolved in SSL, and this liquid was spray-dried to obtain a powdered product. The SSL used in the experiments was produced on the 7th of May 2015 and the LS from the 2nd batch was produced on the 28th of October 2016 at Domsjö Fabriker AB Biorefinery, Örnsköldsvik, Sweden, and kindly provided by Dr. Hans Grundberg. The concentrations of monosaccharides and inhibitors present in the 1st batch of crude SSL were determined by MoRe Research AB (Örnsköldsvik, Sweden) by using High Performance Liquid Chromatography/Ion Chromatography. The concentrations and relative proportions of lignosulphonates, monosaccharides, acetic acid and furfural correspond to those typically found in crude softwood SSL (Björling and Lindman 1989). The final concentrations of monosaccharides and inhibitors present in LBM supplemented with different amounts of SSL were calculated (Table 2).

**Table 2:** Monosaccharide and inhibitor concentrations in crude softwood spent sulphite liquor (SSL) and in LBM supplemented with SSL

Chemical analysis of crude SSL^a^			LBM supplemented with different amounts of crude SSL. Concentrations in g l^−1 b^					
	(g kg^−1^)	(g l^−1^)	5%	10%	15%	20%	25%	30%
Monosaccharides								
Arabinose	0.83	0.92	0.05	0.09	0.14	0.18	0.23	0.27
Galactose	4.35	4.83	0.24	0.48	0.72	0.96	1.20	1.44
Glucose	9.06	10.1	0.50	1.01	1.51	2.02	2.52	3.03
Xylose	10.2	11.3	0.57	1.13	1.69	2.26	2.82	3.39
Mannose	23.1	25.7	1.28	2.57	3.85	5.14	6.42	7.71
Inhibitors								
Formic acid^c^	N/A	1.50	0.08 (0.2)	0.15 (0.5)	0.23 (0.7)	0.30 (1.0)	0.38 (1.20)	0.45 (1.49)
Acetic acid^c^	N/A	4.93	0.25 (2.7)	0.49 (5.3)	0.74 (8.0)	0.98 (10.7)	1.23 (13.4)	1.50 (16.0)
5-HMF^c^	N/A	0.33	0.02	0.03	0.05	0.06	0.08	0.09
Furfural	N/A	0.16	0.01	0.02	0.02	0.03	0.04	0.05
Lignosulfonates	N/A	150	7.5	15.0	22.5	30.0	37.5	45.0

^a^SSL batch no. 20150507. Chemical analysis was performed by MoRE Research (Örnsköldsvik, Sweden)

^b^The concentration of each monosaccharide and inhibitor present in LBM supplemented with different amounts of SSL (%) was calculated from the concentrations of crude SSL obtained through chemical analysis. LBM, Lignin modifying enzyme basal medium (Pointing 1999)

^c^Concentrations of undissociated acid in mM (at pH 4.5) in parenthesis; 5-HMF, 5-(hydroxymethyl)furfural

### Biotolerance to lignosulphonates (LS) and spent sulphite liquor (SSL)

Biotolerance to LS was assessed by monitoring the growth on solid Lilly–Barnett (LiBa) medium (Lilly and Barnett 1951) in Petri dishes (Ø 90 mm), which had been supplemented with 2.5 g of LS l^–1^. Solid LiBa medium was prepared as described for liquid LiBa (Wright et al. 2013), with the addition of 17.8 g bacteriological agar and 898 ml dH_2_O to the solution containing glucose, KH_2_PO_4_ and MgSO_4_·7H_2_O prior to autoclaving, which resulted in a final concentration of 16.0 g bacteriological agar l^–1^. LiBa medium supplemented with 16.0 g bacteriological agar l^–1^ was used as a control. Yeast growth from 48 h-old cultures on solid YPD medium was transferred to LS medium and to solid YPD medium as a positive control. Quadruplicate plates were incubated at 25 °C and growth was assessed visually after one week as + (growth) or − (no growth).

To allow for the simultaneous screening of all yeasts, biotolerance to SSL was tested in two consecutive experiments, a screening experiment and a maximum biotolerance experiment (MBE), in which LBM supplemented with different amounts of SSL and 20.0 g bacteriological agar l^–1^ was used. The pH of the SSL added to the LBM was adjusted to 4.5 with 1 N NaOH from its original pH of 2.7. For the screening experiment, round Petri dishes (Ø 90 mm) were used, and for the MBE, square Greiner Petri dishes (120 × 120 mm) (Sigma-Aldrich, St. Louis, MO, USA) were used. For both biotolerance tests to SSL, yeasts were cultivated overnight at 20 °C on a reciprocal shaker in 15 ml culture tubes, containing 2 ml liquid LBM medium, which had been supplemented with 2.0 g glucose l^–1^. Cells were subsequently centrifuged at 3000 × g and the pelleted cells were resuspended in 1.0 ml of sterile tap water. In the screening experiment, cells from the overnight culture were diluted 50 times in sterile tap water. In the MBE, aliquots of washed cells from the overnight cultures were diluted 20 or 200 times for the count in a haemocytometer, and the remaining suspension of washed cells was diluted in sterile tap water to a final concentration of 4·10^6^ CFU ml^–1^. Aliquots of 5 µl drops of diluted cell suspension were subsequently placed on the LBM agar with various concentrations of SSL: 5%, 10%, 15% or 20% in the screening experiment and 5%, 10%, 15%, 20%, 25% and 30% in the MBE. LBM agar supplemented with 2.0 g glucose l^–1^ and YPD were used as controls. The isolates with tolerance to the highest concentrations of SSL in the screening experiment were selected for further testing in the MBE. In the MBE, an 8-multi channel pipettor was used for applying droplets of cell suspensions to LBM with or without supplementation of SSL. The screening experiment was performed on three separate occasions. The MBE was repeated three times and employed four replicates per plate. Growth was assessed visually as + ,− or w (weak) after incubation of the plates at 20 °C for 72 h. One of the screening experiments was recorded also after 144 h. Results from the MBE were recorded by photography, in addition to visual examination, and photographs were processed in Adobe Photoshop CC 2015 (Adobe Systems Inc. San Jose, Ca, USA).

## Results

In total, 66 isolates of microorganisms were isolated from the chips of decaying wood, retrieved from twelve separate locations on five of the Faroe Islands (Fig. 1). Bacteria predominated, but also some black yeasts were isolated, in addition to the nineteen ascomycetous and basidiomycetous yeasts that are the subject of the present study. Most isolates originated from separate wooden chips, with a few exceptions (Supplementary Table S2). The yeast isolations were concentrated to the central part of the Faroe Islands (Table 1; Fig. 1). Nineteen isolates belonging to eleven different species were identified by sequence comparison of the D1/D2 and ITS sequences. Not all isolates were identified to the species level (Table 1; Supplementary Table S4). Isolates FMYH004 and FBOB002 were identified as *Goffeauzyma gastrica*, FBOC004 as *Holtermanniella takashimae* and FXXA004 as *Naganishia albidosimilis*, respectively, by comparisons of concatenated D1/D2 and ITS sequences, and visualised by phylogenetic trees (Supplementary Figs. S2a–d). Isolate FBOC004 belongs to taxonomic group B of *H. takashimae*, as defined by Wuczkowski et al. (2011) (Supplementary Fig. S2c).

One of the two *Naganishia* strains, *Nag. onofrii* FMYH004b, contained a 9-bp-long insertion within the ITS-sequence. This insertion is present in *Nag. onofrii*, *Naganishia friedmannii* and *Naganishia globosa,* and separates them from other species of *Naganishia*. Within and flanking the insertion, four distinct nucleotide substitutions are present in the sequences of *Nag. onofrii* FMYH004b and *Nag. onofrii* DBVPG5303^T^, a distinguishing characteristic of this species (Turchetti et al. 2015) (Supplementary Fig. S3). These features strengthened the suggested species designation of strain FMYH004b (Supplementary Fig. S2c).

The isolates of *Debaryomyces* and *Rhodotorula* were not possible to classify to the species level by phylogenetic analysis of concatenated D1/D2 and ITS sequences (Supplementary Figs. S4a–b). The sequences of *Debaryomyces* sp. FLYA002 and FTJA004 did not give sufficient information for species assignment. However, the phylogenetic analysis confirms that *Debaryomyces* sp. FLYA002 and FTJA004 do belong to the *Debaryomyces fabryi/hansenii* complex (Groenewald et al. 2008) (Supplementary Table S4; Supplementary Fig. S4a). *Rhodotorula* sp. FMYH002b was found to be closely related to *Rhodotorula araucariae* CBS6031^T^, although the strain with closest resemblance to FMYH002b was *Rhodotorula* sp. PYCC 4824, a strain which initially was designated as *Rhodotorula hamamotoiana* (Supplementary Fig. S4b). This species was never formally described (Coelho et al. 2011), thus *R. hamamotoiana* has remained a ‘*nomen nudum’.*

Most isolates grew in the range of −3 °C – 30 °C. The predominant optimal growth temperature range (OTR) category was that of 15–25 °C, closely followed by that of 15–20 °C. *Rhodotorula* sp. FMYH002b and the two isolates of *Debaryomyces* sp. grew very well at 30 °C, in contrast to other isolates. The most cold-tolerant were isolates of *G. gastrica*, *Cystofilobasidium infirmominiatum, H. takashimae, Candida sake*, *Nadsonia starkeyi-henricii* and *Naganishia albidosimilis*, which grew well at –3 °C. The other isolates also grew at –3 °C, albeit weakly. All isolates were able to grow at a temperature exceeding 20 °C. Hence, they were all classified as psychrotolerant (Table 3).

**Table 3:** Growth temperature preferences of Faroese yeasts and reference strains

Species ^a^	Isolate	Temperature preference (°C)^b^							
		−3	4	15	20	25	30	37	OTR^c^
*Debaryomyces* sp.	FTJA004	w	w	**+**	**+**	**+**	**+**	**−**	20–30
*Debaryomyces* sp.	FLYA002	w	w	**+**	**+**	**+**	**+**	**−**	20–30
*N. starkeyi-henricii*	FAEA002	**+**	**+**	**+**	**+**	**+**	**+**	**−**	15–25
*N. starkeyi-henricii*	FAEA004	**+**	**+**	**+**	**+**	**+**	**+**	**−**	15–25
*C. sake*	FVE002	**+**	**+**	**+**	**+**	**+**	**+**	**−**	15–20
*C. sake*	FNOA002	**+**	**+**	**+**	**+**	**+**	w	**−**	20–25
*C. argentea*	FGAA004	w	**+**	**+**	**+**	**+**	w	**−**	15–25
*Cys. laryngis*	FMYE002	w	w	**+**	**+**	**+**	w	**−**	15–20
*Cys. laryngis*	FMYD002	w	w	**+**	**+**	**+**	w	**−**	15–20
*Cys. laryngis*	FTOF002	w	w	**+**	**+**	**+**	w	**−**	15–25
*Cys. laryngis*	FHVB002	w	w	**+**	**+**	**+**	w	**−**	15–25
*Cyf. infirmominiatum*	FKIA004	**+**	**+**	**+**	**+**	**+**	**−**	**–**	15–25
*Cyf. infirmominiatum*	FTJA008	**+**	**+**	**+**	**+**	**+**	**−**	**−**	15–20
*G. gastrica*	FMYH004	**+**	**+**	**+**	**+**	**+**	**−**	**−**	15–25
*G. gastrica*	FBOB002	**+**	**+**	**+**	**+**	w	**−**	**−**	15–20
*Nag. albidosimilis*	FXXA004	**+**	**+**	**+**	**+**	**+**	**+**	**−**	20–25
*Nag. onofrii*	FMYH004b	w	w	**+**	**+**	**+**	**+**	**−**	15–25
*H. takashimae*	FBOC004	**+**	**+**	**+**	**+**	**+**	w	**−**	15–20
*Rhodotorula* sp.	FMYH002b	w	**+**	**+**	**+**	**+**	**+**	**−**	20–30
*O. thermophila*	CBS11284^T^	**−**	w	w	**+**	**+**	**+**	**+**	30–ND
*Cys. laryngis*	CBS2221^T^	w	w	**+**	**+**	**+**	w	**−**	20–25
*Phe. glacialis*	CBS10436^T^	**+**	w	**+**	**+**	**−**	**−**	**−**	15–15

Reference strains consisted of a thermophilic (*O. thermophila*), a psychrotolerant (*Cys. laryngis*) and a psychrophilic strain (*Phe. glacialis*); + Growth, w Weak Growth, − No Growth, ND Not Determined

^a^*N*., *Nadsonia*; *C*., *Candida*; *Cys*., *Cystobasidium*; *Cyf*., *Cystofilobasidium*; *G*., *Goffeauzyma*; *Nag*., *Naganishia*; *H*., *Holtermanniella; O*., *Ogataea; Phe*., *Phenoliferia*

^b^Growth on YPD agar at 4–37 °C was assessed after six days. Growth at −3 °C was assessed after 18 days

^c^Optimal Growth Temperature Range (OTR) was defined as the temperature range which allowed for profuse growth: Lower and upper cut-off temperatures are indicated

All isolates, except for *H. takashimae* FBOC004, grew well in LiBa medium supplemented with 2.5 g of LS l^−1^ (corresponding to a LS content of 1.7% of SSL). However, this isolate was also unable to grow in pure LiBa medium, suggesting that this was the reason for its absence of growth in LS medium (Table 4).

**Table 4:** Biotolerance to lignosulphonates (LS) and spent sulphite liquor (SSL) in the screening experiment

Species^a^	Isolate	LiBa^b^	LS	Biotolerance (SSL)^d^				
				LBM^c^	5%	10%	15%	20%
*Debaryomyces* sp.	FTJA004	**+**	**+**	**+**	**+**	**+**	**+**	**+**
*Debaryomyces* sp.	FLYA002	**+**	**+**	**+**	**+**	**+**	**+**	**+**
*N. starkeyi-henricii*	FAEA002	**+**	**+**	**+**	w	**−**	**−**	**−**
*N. starkeyi-henricii*	FAEA004	**+**	**+**	**+**	w	**−**	**−**	**−**
*C. sake*	FVE002	**+**	**+**	**+**	**+**	**+**	w	**−**
*C. sake*	FNOA002	**+**	**+**	**+**	**+**	**+**	**+**	**−**
*C. argentea*	FGAA004	**+**	**+**	**+**	**+**	**+**	**+**	**+**
*Cys. laryngis*	FMYE002	**+**	**+**	**+**	**+**	w	w	**−**
*Cys. laryngis*	FMYD002	**+**	**+**	**+**	**+**	w	w	**−**
*Cys. laryngis*	FTOF002	**+**	**+**	**+**	**+**	w	w	**−**
*Cys. laryngis*	FHVB002	**+**	**+**	**+**	**+**	w	w	**−**
*Cyf. infirmominiatum*	FKIA004	**+**	**+**	**+**	**+**	**+**	w	**−**
*Cyf. infirmominiatum*	FTJA008	**+**	**+**	**+**	**+**	**+**	w	**−**
*G. gastrica*	FMYH004	**+**	**+**	**+**	**+**	**+**	**+**	**+**
*G. gastrica*	FBOB002	**+**	**+**	**+**	**+**	w	**−**	**−**
*Nag. albidosimilis*	FXXA004	**+**	**+**	**+**	**+**	w	**−**	**−**
*Nag. onofrii*	FMYH004b	**+**	**+**	**+**	**+**	w	**−**	**−**
*H. takashimae*	FBOC004	**−**	**−**	**+**	**+**	**−**	**−**	**−**
*Rhodotorula* sp.	FMYH002b	**+**	**+**	**+**	**+**	**+**	**+**	**−**
*Cys. laryngis*	CBS2221^T^	ND	ND	**+**	**+**	w	**−**	**−**

Strain *Cys. laryngis* CBS2221^T^ was included as a reference; + Growth, w Weak Growth, − No Growth, ND Not Determined

^a^*N*., *Nadsonia*; *C*., *Candida*; *Cys*., *Cystobasidium*; *Cyf*., *Cystofilobasidium*; *G*, *Goffeauzyma*; *Nag*., *Naganishia*; *H*., *Holtermanniella*

^b^LiBa, Lilly-Barnett medium (Lilly and Barnett 1951)

^c^LBM, Lignin modifying enzyme basal medium (Pointing 1999)

^d^Aliquots of 5 µl of yeast cell suspensions from an overnight culture that had been diluted 1:50 in sterile H_2_O were placed on LBM agar plates containing lignosulphonates. Growth on LBM agar, supplemented with different amounts of spent sulphite liquor (SSL) or with 2.0 g glucose l^−1^ (LBM control) was assessed after 72 h. Growth on LiBa medium or LiBa medium supplemented with 2.5 g of lignosulphonates l^−1^ was assessed after 1 week. The experiment was performed three times. The content of lignosulphonates in the LS medium corresponded to the lignosulphonate concentration in 1.7% SSL

**Fig. 2:**
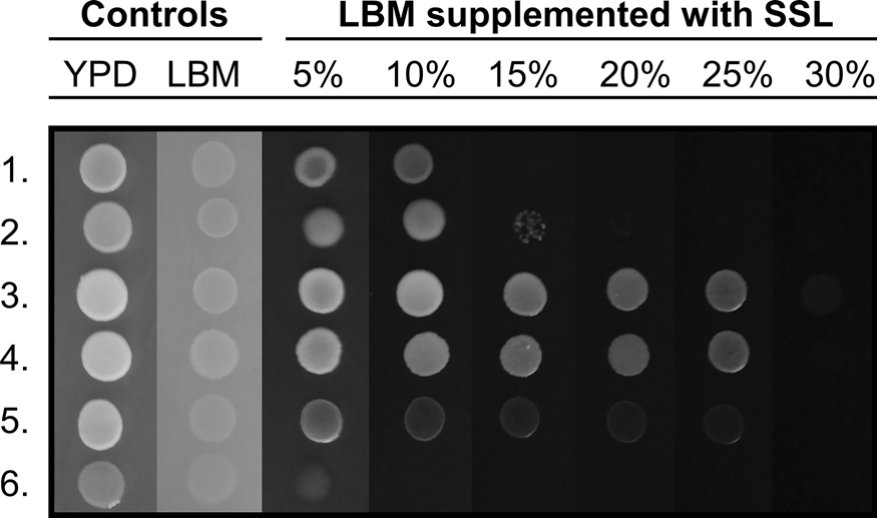
A composite photograph of the MBE (Maximum Biotolerance Experiment), displaying yeast growth that is representative for the three experiments with four replicates in LBM supplemented with increasing amounts of SSL. The most tolerant yeast isolates from the screening experiment were tested. Suspensions of washed yeast cells were added to the LBM media as 5 µl droplets, at a concentration of 4·10^6^ CFU ml^–1^. Growth was recorded after incubation at 20 °C for 72 h. *Cystobasidium laryngis* CBS2221^T^ was included as a reference. 1. *Goffeauzyma gastrica* FMYH004; 2. *Rhodotorula* sp. FMYH002b; 3. *Debaryomyces* sp. FLYA002; 4. *Debaryomyces* sp. FTJA004; 5. *Candida argentea* FGAA004; 6. *Cys. laryngis* CBS2221^T^

All isolates were able to grow in LBM supplemented with 5% SSL as observed in the SSL screening experiment, and all but two isolates (*N. starkeyi-henricii),* grew as well in LBM as in LBM supplemented with 5% SSL. Most isolates also grew in LBM in the presence of 10% SSL. The degree of growth suppression differed among isolates. Six isolates were considered positive for growth in the presence of 15% SSL (Table 4). Growth at 72 h and at 144 h was similar for many of the isolates (data not shown), with the exception of: *Cys. laryngis* FMYD002, FMYE002, FTOF002, FHVB002, *Cyf. infirmominiatum* FTJA008, *Nag. albidosimilis* FXXA004, *Nag. onofrii* FMYH004b and *Rhodotorula* sp. FMYH002b, for which growth had increased during that period. When only the 144 h-observation of the screening experiment was considered, five isolates emerged that could tolerate 20% SSL: *Debaryomyces* sp. FTJA004 and FLYA002, *Candida argentea* FGAA004, *G. gastrica* FMYH004 and *Rhodotorula* sp. FMYH002b. These five isolates were selected for the MBE. The MBE demonstrated that *Debaryomyces* sp. FLYA002, FTJA004 and *C. argentea* FGAA004 grew in LBM medium supplemented with 25% SSL; *Debaryomyces* sp. FLYA002 also grew weakly in the presence of 30% SSL. *G. gastrica* FMYH004 and *Rhodotorula* sp. FMYH002b grew well in concentrations of SSL up to 10% and 15%, respectively (Fig. 2).

## Discussion

The fungal microflora of the Faroe Islands is relatively unexplored. In addition, few studies exist of yeast communities in decaying wood in cold climates (Supplementary Tables S1a–b). The present study investigated the yeasts in decaying wood, isolated from twelve separate geographical locations on the Faroe Islands. All of the yeasts found are frequently documented from polar regions (Buzzini and Margesin 2014), except for *C. argentea* and *R. araucariae* (Holland et al. 2011; Kurtzman et al. 2011a). They are all ubiquitous, however, and not associated with a particular habitat (Kurtzman et al. 2011a). Basidiomycetous yeasts belonging to the Pucciniomycotina and the class Tremellomycetes of the subphyla Agaricomycotina predominated in Arctic basal ice (Butinar et al. 2007). *Cys. laryngis* has been retrieved from several Arctic and Antarctic locations, and it was one of the most abundant members of Pucciniomycotina in samples from Greenland (Butinar et al. 2007; Martinez et al. 2016; Starmer et al. 2005). Similarly, the majority of the yeast isolates in the Faroese collection belonged to Puccinomycotina and Agaricomycotina (specifically, to the Tremellomycetes). *Cyf. infirmominiatum* (formerly *Rhodosporidium infirmominiatum*) and other species of *Cystofilobasidium* are particularly cold-adapted and have been isolated from several cold environments, such as those of Svalbard, Patagonia and Antarctica (Butinar et al. 2007; Cavello et al. 2017; Libkind et al. 2009). Strains of *Goffeauzyma* have been reported from e.g. Iceland, the Alpine region, Russia, Alaska, Svalbard and Antarctica (Białkowska et al. 2017; Butinar et al. 2007; Carrasco et al. 2012; Polyakova et al. 2001; Turchetti et al. 2013; Vishniac 2006). The cold-preference of *G. gastrica* is reflected by the geographical regions from where it is usually isolated and the low maximum temperature of growth (25 °C) (Kurtzman et al. 2011a). This agrees with the results from the present study where the isolates of *Cyf. infirmominiatum* and *G. gastrica* did not grow at temperatures exceeding 25 °C. Moreover, *Nag. albidosimilis* and *Nag. onofrii* are cold-adapted species, isolated from polar and Alpine regions, respectively (Arenz et al. 2006; Butinar et al. 2007; Turchetti et al. 2015). *N. starkeyi-henricii, C. argentea, C. sake*, *R. araucariae* and *H. takashimae* are primarily found in cold and/or temperate climates (Golubev and Pfeiffer 2014; Holland et al. 2011; Kurtzman et al. 2011a; Wuczkowski et al. 2011). *H. takashimae* belongs to the order Holtermanniales, which is related to the orders Filobasidiales and Cystofilobasidiales, which also comprise psychrophilic and psychrotolerant species of the genera *Goffeauzyma*, *Naganishia*, *Filobasidium* and *Mrakia* (Buzzini et al. 2018). In contrast, *Debaryomyces hansenii* is found in many climates (González et al. 1989; Guamán-Burneo et al. 2015; Kurtzman et al. 2011a; Sorenson et al. 1991).

In addition, most of the yeast species reported in the present study are known from maritime climates, except for *C. argentea* and *H. takashimae* (Holland et al. 2011; Wuczkowski et al. 2011).

The Faroese isolates belong to genera and species that are commonly found on plants, in wood, peat and soil. A review of the literature shows that most ascomycetous yeasts from decaying wood in cold and temperate climates are placed in an array of clades belonging to Saccharomycetales; the most prevalent are *Sugiyamaella, Yamadazyma, Kurtzmanniella, Scheffersomyces* and *Debaryomyces* (González et al. 1989; Guamán-Burneo et al. 2015; Middelhoven 2006; Péter et al. 2003; Sorenson et al. 1991)(Supplementary Table S1a). A recent, comprehensive isolation study of ascomycetous yeasts from decaying wood collected at several rainforest sites, demonstrated that Saccharomycetales again dominated, which agrees with the taxonomic position of all ascomycetous yeasts isolated on the Faroe Islands. However, the composition of genera differs in decaying wood from rainforest sites to that from cold and temperate climates (Morais et al. 2020) (Supplementary Table S1a). The yeasts isolated from decaying wood in this study also share similarities to those isolated from decaying Valdivian rainforest wood in Chile during the medium and final stages of wood decay caused by the white-rot fungus *Ganoderma applanatum*. As in the present study, those yeasts belonged to Tremellomycetes and the clades of *Sugiyamaella* and *Scheffersomyces* (González et al. 1989). As in the literature reviewed on yeasts that were isolated from decaying wood in cold and temperate climates (Supplementary Tables S1a–b), the presence of the species *D. hansenii*, *C. sake*, *Cys. laryngis* and *R. araucariae* was confirmed in the present study. However, no previous records of the following six species from decaying wood in cold and temperate climates were found in the reviewed literature: *C. argentea*, *N. starkeyi-henricii, Cyf. infirmominiatum*, *Nag. albidosimilis*, *Nag. onofrii*, *H. takashimae* and *G. gastrica*.

In the present study, *Debaryomyces* sp. FTJA004 and FLYA002 had the highest tolerance to softwood SSL, followed by *C. argentea* FGAA004 and *Rhodotorula* sp. FMYH002b (Fig. 2). Members of the genus *Debaryomyces* (and the family *Debaryomycetaceae*) and the clade of *Rhodosporidium* (such as *Rhodotorula* sp. FMYH002b) are commonly found on decaying wood (Supplementary Table S1a-b). *C. argentea* is probably an unaffiliated Saccharomycetales member (H.-M. Daniel, pers. comm.). The two isolates of *N. starkeyi-henricii* and *H. takashimae* FBOC004 were the least tolerant to SSL (Table 4), and these species are not common on decaying wood (Supplementary Table S1a–b). *N. starkeyi-henricii* is found in soil with organic substrates, whereas *H. takashimae* is associated with plant surfaces (Bourret et al. 2013; Golubev and Pfeiffer 2014; Mestre et al. 2016; Wuczkowski et al. 2011; Yurkov et al. 2012).

Overall, few reports exist of yeast cultivation in SSL. Strains of the following yeast species were cultivated in softwood SSL for the production of ethanol and xylitol: *Meyerozyma guilliermondii*, *Candida tropicalis, Sch. stipitis, Pachysolen tannophilus, Schizosaccharomyces pombe*, *Saccharomyces cerevisiae* and *Scheffersomyces shehatae* (formerly *Candida shehatae*) (Lai and Bura 2012; Lindén and Hahn-Hägerdal 1989; Yu et al. 1987). A strain of *Cyberlindnera jadinii* (formerly *Candida utilis*) was used for single cell protein production in hardwood SSL (Streit et al. 1987). None of these species were represented among the Faroese yeasts. In fact, the Faroese collection consists exclusively of yeast species that previously have not been tested for growth in any type of SLL.

Individual inhibitors have been reported to limit the growth of certain species (Cerrutti and Alzamora 1996; Chen et al. 2009; Delgenes et al. 1996). In the present study, the furan aldehydes and aliphatic acids in SSL were quantified. LBM supplemented with the highest concentration of SSL (30%) contained: 0.09 g 5-HMF l^−1^, 0.05 g furfural l^−1^, 16.0 mM of acetic acid and 1.49 mM of formic acid in undissociated forms at pH 4.5 (Table 2). In order to identify the inhibitors that were growth-limiting to the most superior isolates in SSL (*Debaryomyces* sp. FTJA004 and FLYA002, *C. argentea* FGAA004 and *Rhodotorula* sp. FMYH002b), the inhibitor concentrations mentioned above were compared to reported levels of inhibitor tolerance of related yeasts in the literature.

*D. hansenii* is a fairly robust species, resistant to many stressors (Breuer and Harms 2006), and also to high levels of inhibitors. For example, the growth of *D. hansenii* strain UFV-170 was unaffected by levels of 5-HMF and furfural in 30% SSL (Sampaio et al. 2007). However, the growth of *D. hansenii* strain CCMI-491 was inhibited to 25% by the concentration of acetic acid in 30% SSL (16.0 mM) and to 22% by 1.28 mM of formic acid at pH 4.5 (Duarte et al. 2005). Since the inhibitory effect of acetic and formic acid is additive, i.e. observed inhibition is equal to the sum of the inhibitory effect of each acid (Wang et al. 2013), growth reduction by approximately 25% by either aliphatic acid alone would together have resulted in approximately 50% growth reduction at 30% SSL. The complete absence of growth of *Debaryomyces* sp. isolates FTJA004 and FLYA002 at 30% SSL cannot only be explained by the presence of the aliphatic acids at these concentrations, but was probably due also to additional inhibitors, such as monoaromatic phenolic compounds.

Some strains of Ascomycota of the family Debaryomycetaceae, such as *M. guilliermondii* ATCC 201935 (Kelly et al. 2008) and *Sch. shehatae* ATCC 22984 (Delgenes et al. 1996) were not inhibited by the concentrations of 5-HMF (0.09 g 5-HMF l^−1^) and furfural (0.05 g furfural l^−1^) found in 30% SSL. *C. tropicalis* strain AS 2.1776 showed a growth reduction of 80% at 11.3 mM and no growth at 16.8 mM of undissociated acetic acid (Streit et al. 1987; Wang et al. 2013). A similar growth-inhibition was observed for formic acid. The growth of the same strain of *C. tropicalis* was inhibited to 80% in the presence of 0.89 mM and completely inhibited at 1.68 mM of undissociated formic acid (Wang et al. 2013). Thus, of the four inhibitors investigated, it is probable that acetic acid and formic acid were growth-limiting to the two *Candida* species in the present study. Although some growth of *C. argentea* FGAA004 was observed in the MBE at 25% SSL, it had subsided somewhat already in the presence of 15% SSL (Fig. 2.), which was also the cut-off for growth of *C. sake* FNOA002 and *C. sake* FVE002 in the screening experiment (Table 4).

Yeasts belonging to the *Rhodosporidium* clade of Pucciniomycotina have documented sensitivity to furfural and formic acid. At the concentration of furfural present in 30% SSL (0.05 g furfural l^−1^), growth of *Rhodotorula toruloides* (formerly *Rhodosporidium toruloides*) strain AS 2.1389 was reduced by 20%, with a drastic decline in growth at increasing concentrations. At 0.3 ml furfural l^−1^ (0.35 g furfural l^−1^), growth of *R. toruloides* strain ATCC® 15125^TM^ was reduced by 70% (Zhang et al. 2011). In contrast, 5-HMF resulted in a slight (10%) growth reduction of *R. toruloides* strain AS 2.1389 in the presence of 1.9 g 5-HMF l^−1^, a considerably higher concentration than that in 30% SSL (0.09 g 5-HMF l^−1^) (Hu et al. 2009). Growth of the same strain of *R. toruloides* was only slightly (20%) inhibited by the concentration of undissociated acetic acid present in 30% SSL (16 mM) (Hu et al. 2009). At 0.83 mM undissociated formic acid, growth was reduced by 71% for *Rhodotorula glutinis* strain AS 2.704 (Chen et al. 2009; Hu et al. 2009). Therefore, in the case of *Rhodotorula* sp. FMYH002b, furfural and formic acid appear to have been growth-limiting. Another class of inhibitors present in SSL is the monoaromatic phenolic compounds that are generated through hydrolysis of lignin, e.g. vanillin. The individual concentrations of these compounds in SSL were not determined in the present study. However, in the case of vanillin and syringaldehyde, the levels in crude dilute acid hydrolysate of spruce (*Picea abies*) were determined to be 0.12 g vanillin l^−1^ (0.8 mM) and 0.107 g syringaldehyde l^−1^ (0.6 mM) (Almeida et al. 2007). In a crude dilute acid hydrolysate of *Pinus radiata*, the concentration was found to be 0.4 g vanillin l^−1^ (2.6 mM) (Clark and Mackie 1984). There is limited information on growth of *D. hansenii*, *C. argentea* and *Rhodotorula* sp. in the presence of vanillin, i.e. the most SSL-tolerant yeasts in the present study. The threshold for absence of growth of *D. hansenii* NRRL Y-7239 was somewhere in the interval of 1.0 g vanillin l^−1^ (6.6 mM) to 2.0 g vanillin l^−1^ (13.2 mM) (Cerrutti and Alzamora 1996). Similarly, for *R. toruloides* strain AS 2.1389, it was somewhere between 1.5 g vanillin l^−1^ (9.75 mM) and 2.0 g vanillin l^−1^ (13 mM) (Hu et al. 2009). These growth-inhibitory concentrations of vanillin are thus considerably higher than those reported from acid hydrolysates in the literature. However, the concentrations of total phenolics are relatively high in SSL prepared by acid hydrolysis of spruce or pine: 3.7 g phenolics l^−1^ and 3.35 g phenolics l^−1^, respectively (Almeida et al. 2007; Clark and Mackie 1984). Hence, despite the low concentration of individual monoaromatic phenolic compounds, whose individual effect could be negligible, the concentration of total phenolic monoaromatic compounds in SSL has probably affected the growth of the yeasts in this study. The inhibitory effect of the array of compounds derived from lignocellulosic hydrolysate may act additively if they belong to the same group of compounds and have a similar mode of action (Hu et al. 2009; Kelly et al. 2008; Wang et al. 2013).

An interesting observation is that most species previously reported in studies involving SSL or lignocellulosic hydrolysates belong to the ascomycetous CUG-Ala or CUG-Ser clades, whose enzymes have been shown to possess characteristics related to enhanced fermentation of wood-associated substrates for the production of ethanol and xylitol (Bergmann et al. 2019; Defosse et al. 2018; Krassowski et al. 2018). In the present study, *D. hansenii* and *C. sake* belong to the CUG-Ser clade, which adds to the notion that members of this clade are fit for growth on wood and in SSL.

Most studies on the effect of inhibitors on yeast growth have been carried out for industrial end uses, such as enhancing the production of xylitol, arabinitol, bioethanol, vanillin, plastic monomers, etc. Studies on the effect of inhibitors on yeast growth are therefore limited to a handful of species of commercial importance (mostly within the Saccharomycetes and Schizosaccharomycetes). Thus, many yeast species have never been tested. The suggestion that wood can serve as a reservoir for yeasts with inherent resistance to inhibitors has been explored in studies of strains from rainforest wood, which studied the transformation of xylose in sugarcane bagasse hydrolysate to xylitol (Guamán-Burneo et al. 2015; Morais et al. 2020). The Faroese collection of isolates from decaying wood contained several species of yeasts, which hitherto have no record of cultivation in SSL. Decaying wood in Nordic countries constitutes an untapped resource for retrieving novel yeasts; some clearly hold promise for future valorisation of SSL.

## Supplementary Information

Below is the link to the electronic supplementary material.

Supplementary file1 (DOCX 764 kb)

Supplementary file2 (XLSX 87 kb)

## Data Availability

Not applicable.
