# Supplementary material for: Growth of wood-inhabiting yeasts of the Faroe Islands in the presence of spent sulphite liquor
Source: Antonie Van Leeuwenhoek. 2021 Apr 13;114(6):649–66. doi: 10.1007/s10482-021-01543-5 (PMC8137469; doi:10.1007/s10482-021-01543-5)
Supplement: Supplementary file 1 — Supplementary file1 (DOCX 764 kb) [file 10482_2021_1543_MOESM1_ESM.docx]

**Electronic Supplementary Material – ESM_1 Figures**

Journal: Antonie van Leeuwenhoek

**Growth of wood-inhabiting yeasts of the Faroe Islands in the presence of spent sulphite liquor**

Jonas Rönnander^a^, Sandra Ann Ingela Wright^a^*

^a^Faculty of Engineering and Sustainable Development, University of Gävle, SE-80176 Gävle, Sweden

*Corresponding author: Mailing address for Sandra A. I. Wright: Faculty of Engineering and Sustainable Development, University of Gävle, SE-80176 Gävle, Sweden. Phone: 46-26-648281. E-mail: [sandra.wright@hig.se](mailto:sandra.wright@hig.se)

Fig. S1 Weather data from Tórshavn, Faroe Islands during 2014. Minimum/maximum temperatures (graphs) and monthly precipitation (bars). The arrow indicates the sampling period (9‒12 July, 2014). The temperature ranged between 1 and 13 °C, with an annual average of 11 °C. Data are used with permission from DMI, Danish Meteorological Institute, Copenhagen, Denmark

Fig. S2a‒d Phylogenetic analyses of separate and concatenated ITS1-5.8S-ITS2 and LSU D1/D2 sequences of the Faroese isolates of *Goffeauzyma*, *Holtermanniella* and *Naganishia* for species assignment demonstrate the relatedness to closely-related type-strains. The trees were constructed by using the Maximum Likelihood method based on the Kimura 2-parameter model. Bootstrap analysis was carried out with 1000 replications; values >50% are shown at the nodes.

Fig. S3 A 9-bp-long insertion within the area indicated by the double-headed arrow is characteristic of *Nag. onofrii*, *Nag. friedmannii* and *Nag. globosa,* as described by Turchetti et al. (2015). Molecular similarities and differences within and flanking the 9-bp-long insertion in the ITS-region of *Nag. onofrii*, *Nag. friedmannii* and *Nag. globosa* are indicated in coloured boxes. The boxed bases above the arrowheads indicate the four areas that differentiate *Nag. onofrii* from other species of *Naganishia*. The dashed boxes indicate bases that demonstrate the relatedness of *Nag. onofrii* to *Nag. friedmannii* and to *Nag. globosa,* respectively.

Fig. S4a‒b Phylogenetic analyses of separate and concatenated ITS1-5.8S-ITS2 and LSU D1/D2 sequences of the Faroese isolates of *Debaryomyces* and *Rhodotorula* demonstrate their relatedness to type-strains. The trees were constructed by using the Maximum Likelihood method based on the Kimura 2-parameter model. Bootstrap analysis was carried out with 1000 replications; values >50% are shown at the nodes.

References

**Time of Sampling**

**January**

**February**

**yy**

**Mars**

**April**

**May**

**June**

**July**

**August**

**September**

**October**

**November**

**December**

**MAX**

**MIN**

**(mm)**

**(°C)**

**Fig. S1** Weather data from Tórshavn, Faroe Islands during 2014. Minimum/maximum temperatures (graphs) and monthly precipitation (bars). The arrow indicates the sampling period (9‒12 July, 2014). The temperature ranged between 1 and 13 °C, with an annual average of 11 °C. Data are used with permission from DMI, Danish Meteorological Institute, Copenhagen, Denmark

*Goffeauzyma gastrica* CBS 2288T

99

***Goffeauzyma gastrica* FBOB002**

***Goffeauzyma gastrica* FMYH004**

*Goffeauzyma gilvescens* CBS 7525T

*Naganishia friedmannii* CBS 7160T

0.010

**Fig. S2a** Phylogenetic tree based on the analysis of concatenated ITS and LSU D1/D2 sequences of strains of *Goffeauzyma*. The tree was constructed by using the Maximum Likelihood method based on the Kimura 2-parameter model. Bootstrap analysis was carried out with 1000 replications; values >50% are shown at the nodes. The scale bar indicates the number of substitutions per nucleotide position. *Goffeauzyma gastrica* FMYH004 and *Goffeauzyma gastrica* FBOB002, isolates from the Faroe Islands are indicated in bold.

***Holtermanniella* *takashimae* FBOC004**

84

*Holtermanniella takashimae* CBS 11174^T^

99

*Holtermanniella nyarrowii* CBS 8805^T^

96

*Holtermanniella festucosa* VKM Y-2930^T^

*Holtermanniella wattica* CBS 9496^T^

*Goffeauzyma gastrica* CBS 2288^T^

0.020

**Fig. S2b** Phylogenetic tree based on the analysis of concatenated ITS1-5.8S-ITS2 and LSU D1/D2 sequences of strains of *Holtermanniella*. The tree was constructed by using the Maximum Likelihood method based on the Kimura 2-parameter model. Bootstrap analysis was carried out with 1000 replications; values >50% are shown at the nodes. The scale bar indicates the number of substitutions per nucleotide position. *H. takashimae* isolate FBOC004, isolated from the Faroe Islands, is indicated in bold.

68

61

64

MK045394.1 *Holtermanniella takashimae* IOM 325336, crater lake, Mexico

AY749434.1 *Cryptococcus* sp. YKS 2004, liquid nitrogen vessel, South Korea

MG589202.1 *Holtermanniella takashimae* HU-9215, lettuce, USA

FM246501.1 *Holtermanniella takashimae* CBS 11174T, plant litter, Austria (type-strain)

HG532069.1 *Cryptococcus* sp. R46, barley, Finland

KM384182.1 *Holtermanniella takashimae*  yHKS124, soil, USA

KU728172.1 *Holtermanniella takashimae* L8.12, *Vaccinium*, Patagonia (Argentina)

FN430732.1 *Cryptococcus* sp. CRUB1154, grassland soil, Germany

KX067814.1 *Holtermanniella takashimae* RP294, beans, Germany

**MK737520.1 *Holtermanniella takashimae* FBOC004, decaying wood, The Faroe Islands**

HG936759.1 Uncultured *Cryptococcus* sp., *Zea mays* field bulk soil, Germany

AM901778.1 Uncultured basidiomycete, house dust, Finland

KM816683.1 *Holtermanniella nyarrowii* LKF08-137, ice-covered lake, Antarctica

68

61

64

MK045394.1 *Holtermanniella takashimae* IOM 325336, crater lake, Mexico

AY749434.1 *Cryptococcus* sp. YKS 2004, liquid nitrogen vessel, South Korea

MG589202.1 *Holtermanniella takashimae* HU-9215, lettuce, USA

KU728172.1 *Holtermanniella takashimae* L8.12, *Vaccinium*, Patagonia (Argentina)

FN430732.1 *Cryptococcus* sp. CRUB1154, grassland soil, Germany

KX067814.1 *Holtermanniella takashimae* RP294, beans, Germany

**MK737520.1 *Holtermanniella takashimae* FBOC004, decaying wood, The Faroe Islands**

HG936759.1 Uncultured *Cryptococcus* sp., *Zea mays* field bulk soil, Germany

AM901778.1 Uncultured basidiomycete, house dust, Finland

KM816683.1 *Holtermanniella nyarrowii* LKF08-137, ice-covered lake, Antarctica

MN913531.1 *Holtermanniella takashimae* AD10, wheat, Italy

JX188168.1 *Holtermanniella takashimae* P34B009, vineyard, USA

**B**

**A**

0.0050

FM246501.1 *Holtermanniella takashimae* CBS 11174^T^, plant litter, Austria (type-strain)

HG532069.1 *Cryptococcus* sp. R46, barley, Finland

KM384182.1 *Holtermanniella takashimae*  yHKS124, soil, USA

**Fig. S2c** Phylogenetic tree, demonstrating the relationship of strains of *Holtermanniella* to *H. takashimae* isolate FBOC004 by using analysis of ITS1-5.8S-ITS2 sequences. The tree was constructed by using the Maximum Likelihood method based on the Kimura 2-parameter model. Bootstrap analysis was carried out with 1000 replications; values >50% are shown at the nodes. *H. takashimae* isolate FBOC004 from the Faroe Islands is indicated in bold. Accession numbers are indicated to the left. The letters **A** and **B** correspond to the groups that were defined by Wuczkowski et al. (2011).

*Naganishia albidosimilis* CBS7711^T^

100

***Naganishia albidosimilis* FXXA004**

99

0.020

*Naganishia diffluens* CBS 160^T^

0.020 *Naganishia diffluens* CBS 160^T^

94

*Naganishia liquefaciens* CBS 968^T^

99

*Naganishia liquefaciens* CBS 968^T^

*Naganishia adeliensis* CBS 8351^T^

97

*Naganishia albida* CBS 142^T^

*Naganishia globosa* CBS 1975^T^

*Naganishia globosa* CBS 1975^T^

*Naganishia friedmannii* CBS 7160^T^

100

*Naganishia friedmannii* CBS 7160^T^

52

*Naganishia onofrii* DBVPG 5303^T^

98

***Naganishia onofrii* FMYH004b**

*Goffeauzyma gastrica* CBS 2288^T^

**Fig. S2d** Phylogenetic tree based on the analysis of concatenated ITS1-5.8S-ITS2 and LSU D1/D2 sequences of strains of *Naganishia*. The tree was constructed by using the Maximum Likelihood method based on the Kimura 2-parameter model. Bootstrap analysis was carried out with 1000 replications; values >50% are shown at the nodes. The scale bar indicates the number of substitutions per nucleotide position. *Nag. albidosimilis* FXXA004 and *Nag.onofrii* FMYH004b, isolates from the Faroe Islands are indicated in bold.

9-bp insertion


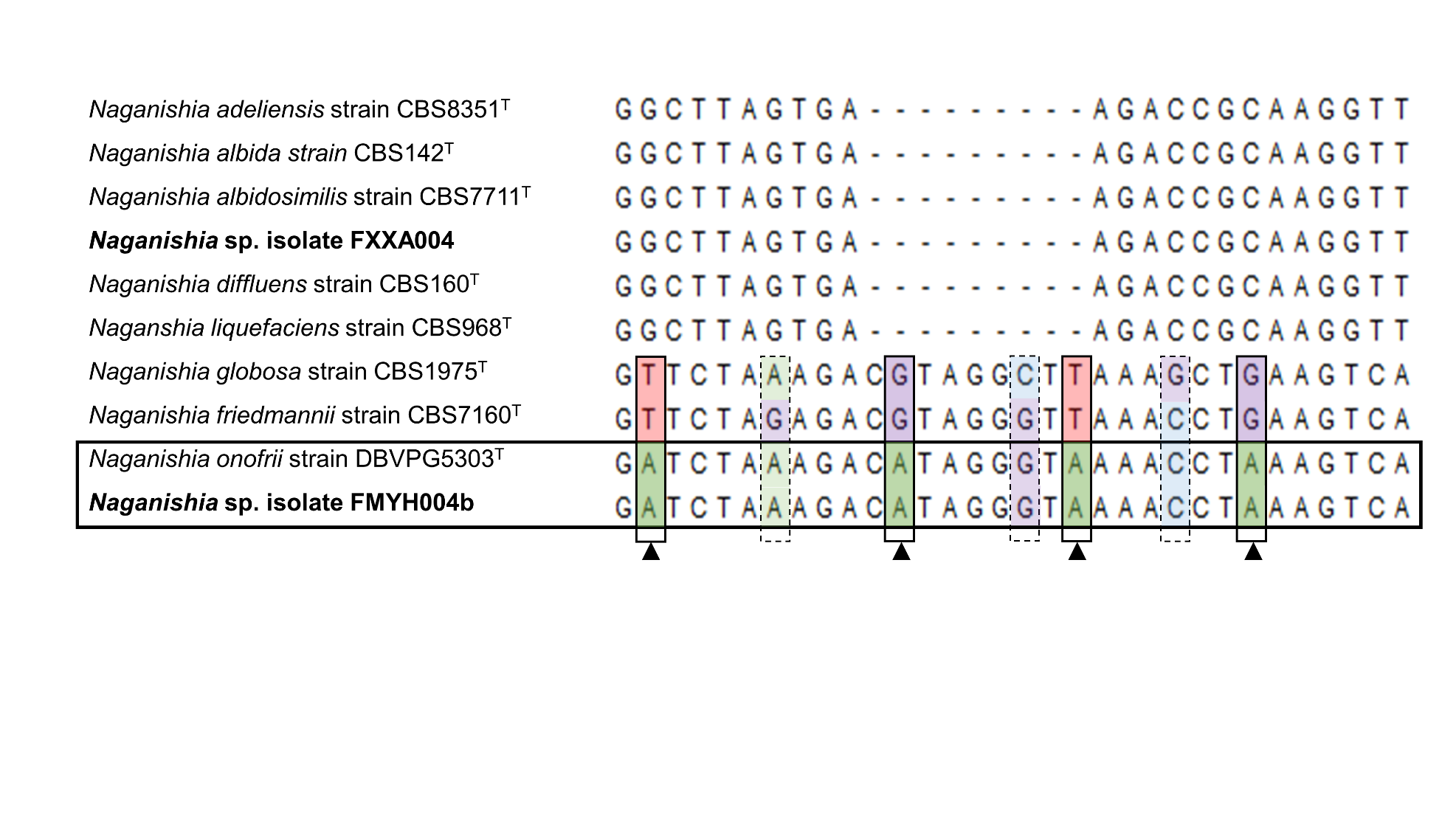


***Naganishia onofrii* FMYH004b**

*Naganishia onofrii* DBVPG5303^T^

*Naganishia friedmannii* CBS7160^T^

*Naganishia globosa* CBS1975^T^

*Naganishia liquefaciens* CBS968^T^

*Naganishia diffluens* CBS160^T^

***Naganishia albidosimilis* FXXA004**

*Naganishia albidosimilis* CBS7711^T^

*Naganishia albida* CBS142^T^

*Naganishia adeliensis* CBS8351^T^

**Fig. S3** A 9-bp-long insertion within the area indicated by the double-headed arrow is characteristic of *Nag. onofrii*, *Nag. friedmannii* and *Nag. globosa,* as described by Turchetti et al. (2015). Molecular similarities and differences within and flanking the 9-bp-long insertion in the ITS-region of *Nag. onofrii*, *Nag. friedmannii* and *Nag. globosa* are indicated in coloured boxes. The boxed bases above the arrowheads indicate the four areas that differentiate *Nag. onofrii* from other species of *Naganishia*. The dashed boxes indicate bases that demonstrate the relatedness of *Nag. onofrii* to *Nag. friedmannii* and to *Nag. globosa,* respectively.

64

**B (D1/D2)**

**A (ITS)**

**MK749417.1 *Debaryomyces* sp. FTJA004**

KY107531.1 *Debaryomyces hansenii* var. *hansenii* CBS767^T^

KY107591.1 *Debaryomyces vindobonensis* CBS11666^T^

NG_055701.1 *Debaryomyces prosopidis* JCM 9913^T^

**MK749768.1 *Debaryomyces* sp. FLYA002**

NG_055699.1 *Debaryomyces subglobosus* CBS792^T^

FR693385.1 *Debaryomyces* sp. CLIB1277^T^ ***Clade A***

U94927.1 *Debaryomyces fabryi* CBS789^T^

KY107556.1 *Debaryomyces hansenii* CBS1795 ***Clade B***

KY107578.1 *Debaryomyces nepalensis* CBS5921^T^

AF210327.1 *Debaryomyces hansenii* var*. hansenii* CBS767^T^

NR_138218.1 *Debaryomyces vindobonensis* CBS 11666^T^

NR_077067.1 *Debaryomyces prosopidis* JCM 9913^T^

**MK737678.1 *Debaryomyces* sp. FLYA002**

FN675240.1 *Debaryomyces subglobosus* CBS792^T^

FR693386.1 *Debaryomyces* sp. CLIB1277^T^ ***Clade A***

NR_138186.1 *Debaryomyces fabryi* CBS789^T^

KY103258.1 *Debaryomyces hansenii* CBS1795 ***Clade B***

KY103281.1 *Debaryomyces nepalensis* CBS5921^T^

0.0010

0.0010

**Fig. S4a** Phylogenetic tree based on the analysis of ITS1-5.8S-ITS2 (**A**) and LSU D1/D2 (**B**) sequences of strains of *Debaryomyces*. The tree was constructed by using the Maximum Likelihood method based on the Kimura 2-parameter model. Bootstrap analysis was carried out with 1000 replications; values >50% are shown at the nodes. The scale bar indicates the number of substitutions per nucleotide position. *Debaryomyces* sp. FTJA004 and *Debaryomyces* sp. FLYA002, isolates from the Faroe Islands are indicated in bold. Accession numbers are indicated to the left of the strains within the phylogenetic trees. Clade A and B, defined by Jacques et al. (2015), are indicated in colour.

*Rhodotorula* sp. PYCC 4824 "hamamotoiana"

91

81 ***Rhodotorula* sp. FMYH002b**

*Rhodotorula araucariae* CBS 6031T

*Rhodotorula kratochvilovae* CBS 7436T

*Rhodotorula glutinis* CBS 20T

0.0050

**Fig. S4b** Phylogenetic tree based on the analysis of concatenated ITS1-5.8S-ITS2 and LSU D1/D2 sequences of strains of *Rhodotorula*. The tree was constructed by using the Maximum Likelihood method based on the Kimura 2-parameter model. Bootstrap analysis was carried out with 1000 replications; values >50% are shown at the nodes. The scale bar indicates the number of substitutions per nucleotide position. *Rhodotorula* sp. FMYH002b, isolated from the Faroe Islands, is indicated in bold.

**References**

Jacques N, Zenouche A, Gunde-Cimerman N, Casaregola S (2015) Increased diversity in the genus *Debaryomyces* from Arctic glacier samples. Antonie van Leeuwenhoek 107:487-501. https://doi.org/10.1007/s10482-014-0345-7

Turchetti B, Selbmann L, Blanchette RA, Di Mauro S, Marchegiani E, Zucconi L, Arenz BE, Buzzini P (2015) *Cryptococcus vaughanmartiniae* sp. nov. and *Cryptococcus onofrii* sp. nov.: two new species isolated from worldwide cold environments. Extremophiles 19:149-159. https://doi.org/10.1007/s00792-014-0692-3

Wuczkowski M, Passoth W, Turchetti B, Andersson A-C, Olstorpe M, Lairila A, Theelen B, van Broock M, Buzzini P, Prillinger H, Sterflinger K, Schnürer J, Boekhout, T, Libkind D (2011) Description of *Holtermanniella* gen. nov., including *Holtermanniella takashimae* sp. nov. and four new combinations, and proposal of the order Holtermanniales to accommodate tremellomycetous yeasts of the *Holtermannia* clade. Int J Syst Evol Micr 61:680-689. https://doi.org/10.1099/ijs.0.019737-0

Turchetti B et al. (2015) Cryptococcus vaughanmartiniae sp. nov. and Cryptococcus onofrii sp. nov.: two new species isolated from worldwide cold environments Extremophiles 19:149-159 doi:10.1007/s00792-014-0692-3
